# Anti-Tumor Effect of Rutin on Human Neuroblastoma Cell Lines through Inducing G2/M Cell Cycle Arrest and Promoting Apoptosis

**DOI:** 10.1155/2013/269165

**Published:** 2013-12-29

**Authors:** Hongyan Chen, Qing Miao, Miao Geng, Jing Liu, Yazhuo Hu, Lei Tian, Jingkun Pan, Yi Yang

**Affiliations:** ^1^Beijing Key Laboratory for Aging and Geriatrics, Institute of Geriatrics, General Hospital of Chinese PLA, Beijing, China; ^2^National Institute of Occupational Health and Poison Control, Chinese Center for Disease Control and Prevention, Beijing, China; ^3^Minzu University of China, Beijing, China

## Abstract

*Aims*. To further investigate the antineuroblastoma effect of rutin which is a type of flavonoid. *Methods*. The antiproliferation of rutin in human neuroblastoma cells LAN-5 were detected by 3-(4,5-dimethylthiazol-2-yl)-2,5-diphenyltetrazolium bromide (MTT) assay. Chemotaxis of LAN-5 cells was assessed using transwell migration chambers and scratch wound migration assay. The cell cycle arrest and apoptosis in a dose-dependent manner was measured by flow cytometric and fluorescent microscopy analyses. The apoptosis-related proteins BAX and BCL2 as well as MYCN mRNA express were determined by RT-PCR analysis. Secreted TNF-**α** level were determined using specific enzyme-linked immunosorbent assay kits. *Results*. Rutin significantly inhibited the growth of LAN-5 cells and chemotactic ability. Flow cytometric analysis revealed that rutin induced G2/M arrest in the cell cycle progression and induced cell apoptosis. The RT-PCR showed that rutin could decrease BCL2 expression and BCL2/BAX ratio. In the meantime, the MYCN mRNA level and the secretion of TNF-**α** were inhibited. *Conclusion*. These results suggest that rutin produces obvious antineuroblastoma effects via induced G2/M arrest in the cell cycle progression and induced cell apoptosis as well as regulating the expression of gene related to apoptosis and so on. It supports the viability of developing rutin as a novel therapeutic prodrug for neuroblastoma treatment, as well as providing a new path on anticancer effect of Chinese traditional drug.

## 1. Introduction

Neuroblastoma (NB) is the most common extracranial solid tumor in childhood, and it is the most common type of cancer to be diagnosed in the first year of life [1]. While the overall survival rate for children with low- and intermediate-risk NB has been consistently improved, less than 40% of high-risk NB patients survive, in spite of the intensification of the multiagent induction therapy, along with surgery [2]. Consequently, further advances in therapy are necessary to target NB tumor cells in a more efficient way to gain clinical benefits without substantially increasing toxicity.

**Figure 1:**
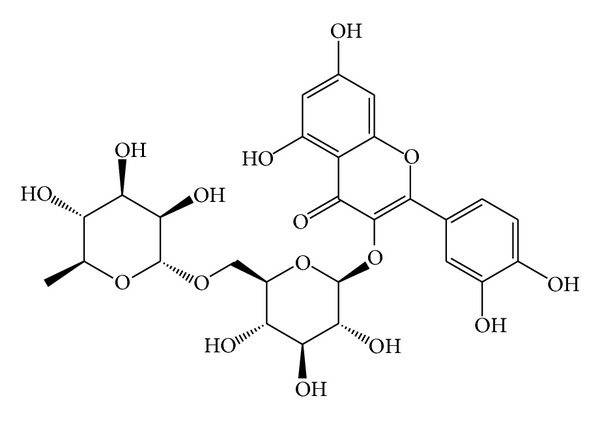
The chemical structure of rutin.

The first recorded use of herbs for medical treatment begun 4000 years ago, originating from China and India. Recently, increasing attention has been focused on the application of herbs in tumor therapy all over the world. Traditional Chinese medicine has multieffects, being immunomodulatory and anti-inflammatory as well as inducing apoptosis; it may also function as radiosensitizers during the radiotherapy of cancer [3]. Advantages of Chinese medicine are providing treatment method for Patients who lost surgical and chemotherapy opportunity. Flavonoids are polyphenolic compounds that are ubiquitously in plants. The flavonoids have aroused considerable interest recently because of their potential beneficial effects on human health; they have been reported to have antiviral, antiallergic, antiplatelet, anti-inflammatory, antitumor, and antioxidant activities [4–6]. In a 20-year study, it was found that participants with the highest intake of flavonoids and proanthocyanidins had a 44 and 40 percent lower risk of oral cancer and laryngeal cancer, respectively, and the occurrence of colon cancer was also lowered by a third, and there were reductions in breast, kidney, and ovarian cancers too [7]. Based on these results, flavonoids may be promising anticancer agents. Rutin (quercetin rutinoside) (Figure 1) is a glycoside of the flavonoid quercetin. Many beneficial effects of rutin have been identified, including inhibition of platelet aggregation, being anti-inflammatory, antioxidant, and reduction of blood fat and cholesterol [8]. The auticancer research showed that rutin could exert significant and potentially beneficial effects on decreasing the amount of precancerous lesions and inducing apoptosis in the large intestine cancer [9], but for the treatment of neuroblastoma effect has not been reported. Therefore, we investigated whether rutin could couse anti-proliferation and induction of apoptosis in human neuroblastoma LAN-5 cells. The results show that rutin produces obvious antineuroblastoma effects via induced G2/M arrest in the cell cycle progression and induced cell apoptosis as well as regulating the expression of gene related to apoptosis.

## 2. Experimental Procedures

### 2.1. MTT Assay

Cultured LAN-5 cells (the human neuroblastoma cell strain was graciously provided by the Academy of Military Medical Sciences) were digested, suspended in DMEM medium supplemented with 10% FBS, counted, and inoculated in a 96-well plate at a concentration of 1 × 10^4^ cells per well. One day, after inoculation, the adherent cells were cultured in serum-free DMEM and pretreated with either 0, 25, 50 *μ*M, or 100 *μ*M rutin (Rutin with purity >92.5% was purchased from the National Institute for the Control of Pharmaceutical and Biological Products) for 24 h. The culture media were collected and stored at −20°C for future use, and the cells were reacted with 5 mg/mL 3-(4,5-dimethylthiazol-2-yl)-2,5-diphenyltetrazolium bromide (MTT) solution for 4 h. The reaction was terminated by adding 100 *μ*L DMSO, and the absorbance at 570 nm was determined using an enzyme-linked immunosorbent assay reader.

### 2.2. Cell Cycle Analysis

Flow cytometric cell cycle analysis was performed as described previously. A density of 1 × 10^6^ cells/well were seeded into 6-well plates and left to attach overnight, similar to MTT assay. The cells were incubated with 0, 25, 50, and 100 *μ*M of rutin for 24 h. After incubation, cells were harvested and washed with phosphate buffered saline (PBS), resuspended, and fixed in 75% ice-cold ethanol overnight at 4°C. Then the cells were treated with RNase A (50 mg/mL) and propidium iodide (PI, Sigma) (50 mg/mL) and analyzed by the Coulter Epics XL Flow Cytometer (Beckman, USA).

### 2.3. Apoptosis Detection with Flow Cytometry (Double Staining with Annexin V-FITC/PI)

The double dye Annexin V-FITC/PI was used to distinguish living cells, early and late apoptosis cells, and necrotic cells. LAN-5 cells were treated with different concentrations of rutin for 24 h as previously described, then harvested and washed with PBS, and then resuspended in the PBS. Cells were stained with Annexin V-FITC/PI according to the protocol of Annexin V-FITC cell Apoptosis Detection Kit. After incubation, the samples were pelleted to the Coulter Epics XL Flow Cytometer (Beckman, USA).

### 2.4. Cytokine Analysis

The collected supernatants from each treatment were incubated at room temperature and were used to measure TNF-*α* level using specific enzyme-linked immunosorbent assay (ELISA) kits, according to the manufacturer's instruction. The TNF-*α* cytokine levels are shown as the mean ± SD (picograms of each cytokine per milliliter).

### 2.5. RT-PCR Assay

LAN-5 cells were treated and collected, and total RNA was extracted using the mRNA extraction kit (Beijing Tiandz Gene Engineering, Beijing, China). Nucleic acid concentration was determined using a UV spectrophotometer (Shimadzu, Kyoto, Japan). A total of 2 *μ*g total RNA was harvested and reverse-transcribed and synthesized into cDNA according to Fermentas reverse transcriptase kit instructions (Vilnius, Lithuania). A total of 2 *μ*L reverse transcription product was amplified using specific primers: *BCL2*: forward 5′-TGTGGCCCAGATAGGCACCCAG-3′ and reverse 5′-ACTTCGCCGAG ATGTCCAGCCAG-3′, product 370 bp: *BAX*: forward 5′-ACCAAGAAGCTGAGCGAGTATC-3′ and reverse 5′-ACAAAGATGGTCACGGTCTGCC-3′, product 367 bp; MYCN: forward, 5′-CTCAGTACCTC CGGAGAG-3′ and reverse, 5′-GGCATCGTTTGAGGATC-3′ product 177 bp; GAPDH: forward 5′-GCCAAAAGGG TCATCA TCTC-3′ and reverse 5-GGCCATCCACAGTCTTCT-3′ product 196 bp. For BCL2 PCR conditions were 5 min at 94°C, amplification for 30 cycles with 1 min at 94°C, 1 min at 58°C and a final extension for 1 min at 72°C; For BAX PCR conditions were5 min at 94°C, amplification for 30 cycles with 1 min at 94°C, 1 min at 60°C and a final extension for 1 min at 72°C; For MYCN PCR conditions were 5 min at 94°C, amplification for 30 cycles with 1 min at 94°C, 1 min at 60°C and a final extension for 1 min at 72°C; For GAPDH PCR conditions were5 min at 94°C, amplification for 30 cycles with 1 min at 94°C, 1 min at 56°C and a final extension for 1 min at 72°C. Products were separated by 2.0% agarose gel electrophoresis and photographed using a gel imaging analysis system (Shanghai Talent Electronics, Shanghai, China).

### 2.6. Scratch Wound Migration Assay

A density of 1 × 10^5^ cells/well were seeded into 6-well plates; cells were cultured for approximately 24 h, at which time they were approximately 80% confluent. The monolayer was scratched with a sterile 200 *μ*L pipette tip, and the cells were treated with rutin (0, 25, 50, 100 *μ*M) then incubated for a further 48 h to allow time for migration into the cell-free area.

### 2.7. Transwell Assay

Chemotaxis in LAN-5 cells was assessed using transwell migration chambers (8 *μ*m pore polycarbonate filters in 24 wells). LAN-5 cells were plated into the top wells (6 × 10^4^ cells per well), which were filled with medium alone or in absence of rutin (25 *μ*M, 50 *μ*M, and 100 *μ*M) and incubated for 24 h at 37°C. Cells on the top side of the filter were wiped off using a cotton bud, and cells on the lower surface of the filter were fixed in 100% methanol for 3 min and subjected to 4′,6-diamidino-2-phenylindole (DAPI) staining. Then, the cells were counted under a fluorescence microscope (5 fields were examined for each condition).

### 2.8. Statistical Analysis

All the data were normally distributed; therefore, in instances of single mean comparisons, Levene's test for equality of variances followed by a Student's *t*-test for independent samples was used to assess significance. In instances of multiple mean comparisons, ANOVA was used, followed by post hoc comparisons using Bonferroni's method. The levels were set at 0.05 for all the analyses. The statistical package for the social sciences release 19 (SPSS, SN: 5087722) was used for all the data analyses.

## 3. Results

### 3.1. Effect of Rutin on Cell Viability of LAN-5 Cells

**Figure 2:**
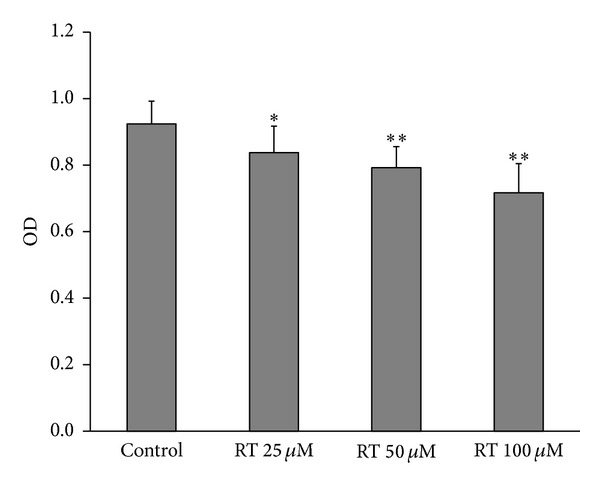
The effect of rutin on cell viability. Cells were treated with rutin (RT 25 *μ*M, RT 50 *μ*M, or RT 100 *μ*M) for 24 h. Data are shown as the means of three experiments. *P < 0.05; **P < 0.01 compared to control.

We first examined the effects of rutin on the viability of LAN-5 cells. The MTT assay (Figure 2) showed that rutin treatment significantly inhibited the viability of LAN-5 cells in a dose dependent manner, OD values from 0.92 ± 0.06 to 0.83 ± 0.07 and 0.79 ± 0.06 as well as 0.71 ± 0.08, respectively.

### 3.2. Rutin Induced Cell Cycle Arrest in G2/M Phase in LAN-5 Cells

**Figure 3:**
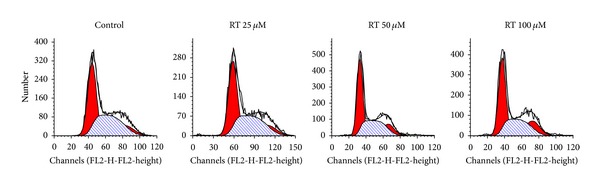
The effect of rutin on cell cycle. Cells were treated with rutin for 24 h with designated rutin then washed with PBS to remove complete medium, and fixed with 75% per-cold alcohol then stained with PI, and the DNA content was analyzed by flow cytometry.

The effects of rutin on cell cycle progress in LAN-5 cells, were investigated by flow cytometry. Cells incubated with different concentrations (0, 25, 50, and 100 *μ*M) of rutin for 24 h were analyzed for the distribution of G0/G1, S, and G2/M phases of cell cycle (Figure 3). Compared with control, rutin treatment resulted in a significant accumulation of cells in the G2/M phase, the percentage of LAN-5 cells in the G2/M fraction increased from 8.8% to 10.05%, 14.46%, and 20.05% after treated with 25, 50, and 100 *μ*M of rutin (Table 1), which underwent G2/M arrest in a dose-dependent way.

### 3.3. Rutin Induced LAN-5 Apoptosis Is Mediated by Induction of BCL2/BAX Imbalance

**Figure 4:**
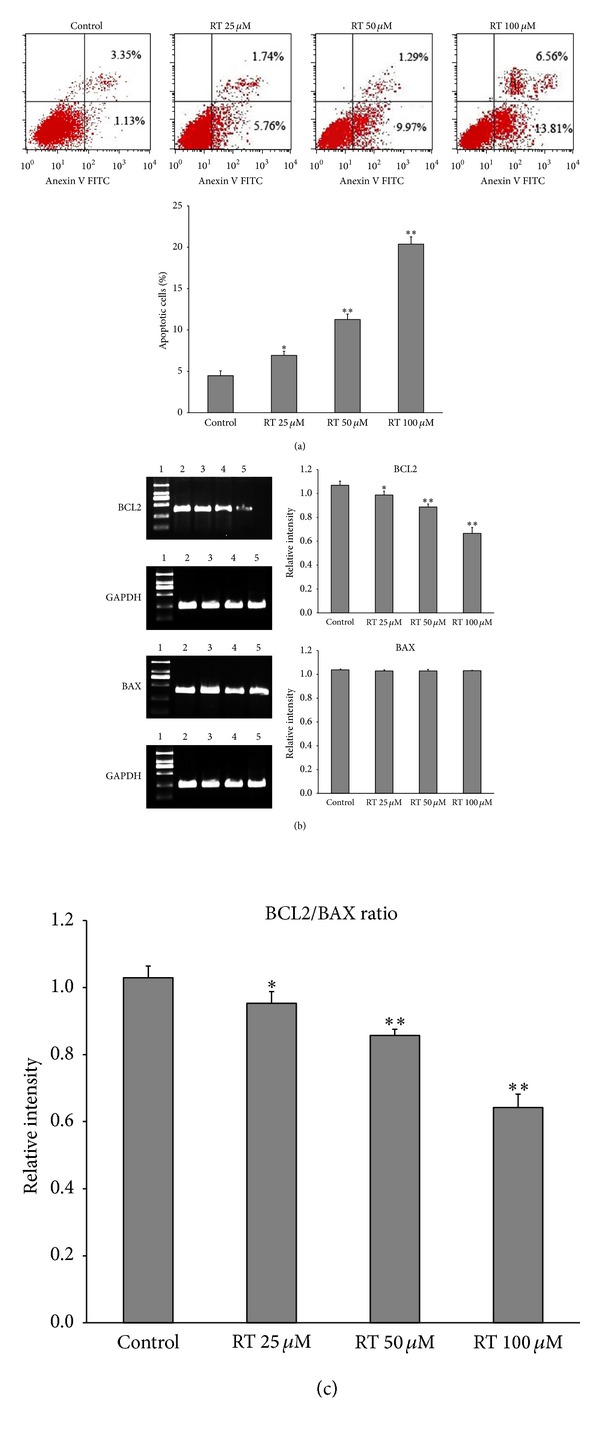
The effect of rutin on cell apoptosis and expression of BCL2 and BAX. (a) Apoptosis rates of LAN-5 incubated with different concentrations of rutin, the percentage of apoptotic cells were measured by flow cytometry using the PI-annexin V assay. (b) Quantification of BCL2 and BAX mRNA levels, (c) calculated BCL2/BAX ratios. The “relative intensity” was defined as the intensity (target mRNA)/intensity (GAPDH). All experiments were repeated three times. *P < 0.05,**P < 0.01 compared to control. 1: marker; 2: control; 3: RT 25 *μ*M; 4: RT 50 *μ*M; 5: Rt 100 *μ*M.

Annexin V-FITC/PI apoptosis assay kit was used to detect cell apoptosis (Figure 4(a)). Normal cells were used as the control group. It seems that the addition of different dose rutin induced apoptosis of LAN-5 cells with percentages of apoptosis cells increasing from 4.48% to 7.50%, 11.26%, and 20.37%. Meanwhile, RT-PCR analysis showed that the expression of BCL2 was downregulated by rutin and BCL2/BAX ratios were downregulated (Figures 4(b) and 4(c)).

### 3.4. Effect of Rutin on TNF-*α* Release in LAN-5 Cells

**Figure 5:**
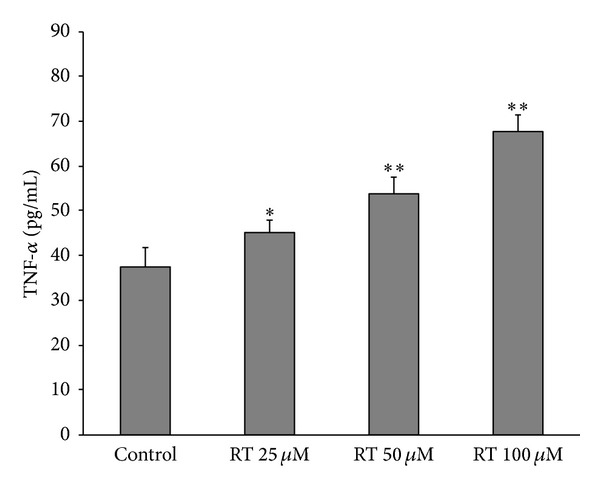
The effects of rutin on TNF-*α* secretion. The results of ELISA measuring TNF-*α* contents in the supernatants of LAN-5 cells treated with 0, 25, 50 and 100 *μ*M rutin (RT 25 *μ*M, RT 50 *μ*M or RT 100 *μ*M) for 24 h. Data shown are the means of three experiments. *P < 0.05; **P < 0.01 compared to control.

We further explored the effect of rutin on the secretion of TNF-*α* in LAN-5 cells. As shown (Figure 5), treatment with 25 *μ*M and 50 *μ*M or 100 *μ*M rutin markedly increased TNF-*α* secretion from 37.59 ± 4.17 to 44.97 ± 2.94 and 53.63 ± 4.0 as well as 67.72 ± 3.89 pg/mL, respectively.

### 3.5. Effect of Rutin on Cell Migration and Invasion Capability

**Figure 6:**
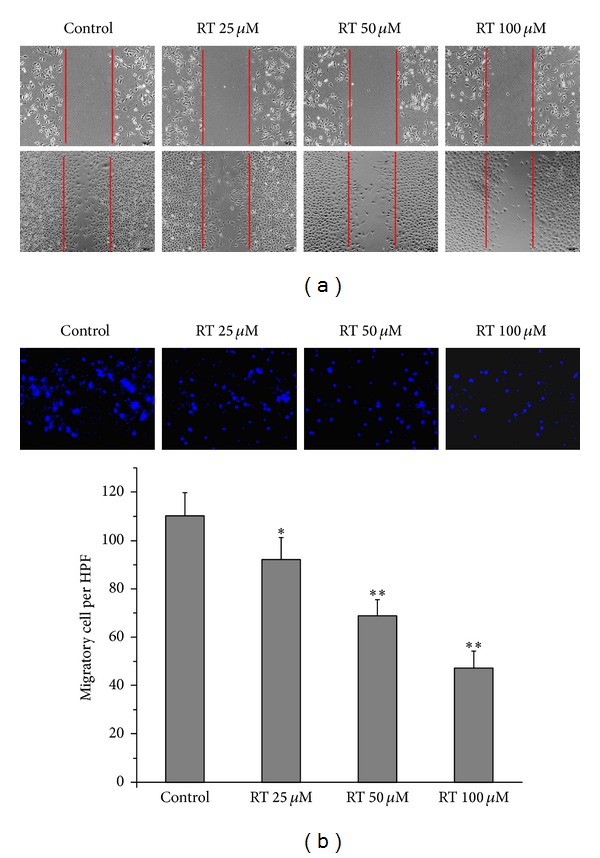
Effect of rutin on cell migration and invasion capability. The representative pictures in cell wounding and migration assay. (a) The pictures show the migration of LAN-5 cells in the martrigel surface after 48 hr of scrape in different groups. The dot line shows the initial start point where the cell began the migration process. (b) The pictures show the transwell assay. The cells that have migrated through the pores to the lower surface of the membrane were counted under microscope. All experiments data are expressed as means ± SEM, of the three measurements *P < 0.05; **P < 0.01 compared to control.

Cell monolayer wounding and migration test were performed to examine the role of rutin on cell migration. LAN-5 cells were seeded in 6-well plates; wounds were created by scrapping the cell monolayer by P200 pipette tips. As shown in Figure 6(a), different dose rutin could decrease the migration rate of LAN-5 cells, as compared to that of control LAN-5 cells, indicating that rutin is involved in migration capability of LAN-5 cells. Matrigel invasion assay was performed to examine the role of rutin in LAN-5 cell invasion. LAN-5 cells were seeded on the upper chamber of transwells coated with matrigel. After 24 hours, the number of cells that invaded to the lower chamber is significantly lower in different dose rutin groups compared to the control group (*P* < 0.05, *P* < 0.01; Figure 6(b)), indicating that rutin suppressed the invasion abilities. These results indicated that the rutin plays an important role in the invasion and migration capability of LAN-5 cells.

### 3.6. Effect of Rutin on MYCN Expression in LAN-5 Cells

**Figure 7:**
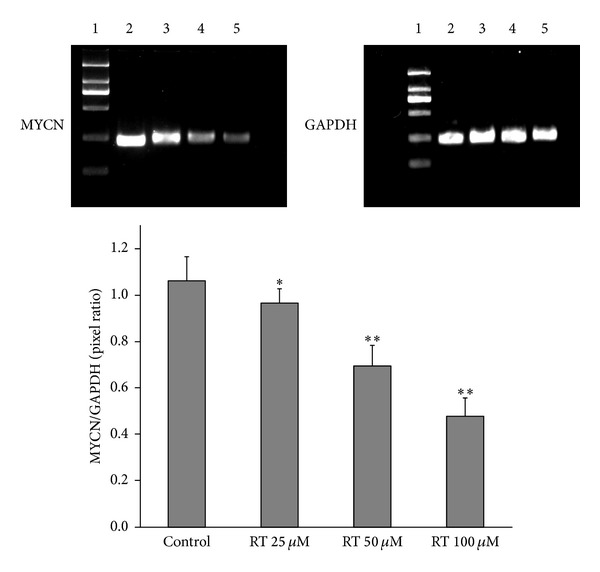
The effect of rutin on expression of MYCN. MYCN mRNA expression is high in normal cells, rutin significantly reduces MYCN mRNA expression in LAN-5. *P < 0.05; **P < 0.01 compared to control. 1: marker; 2: control; 3: RT 25 *μ*M; 4: RT 50 *μ*M; 5: RT 100 *μ*M.

Overexpression of MYCN in neuroblastoma has been correlated with treatment failure; the inhibition of MYCN expression may prove to be a useful target for treatment of neuroblastoma. High MYCN mRNA expression was observed in LAN-5 cells (MYCN/GAPDH ratio was 1.06 ± 0.10). The different doses of rutin treatment significantly reduced MYCN mRNA expression in the LAN-5 cells (MYCN/GAPDH ratios were 0.96 ± 0.06, 0.69 ± 0.09, and 0.48 ± 0.08) (Figure 7).

## 4. Discussion

In China, traditional Chinese medicine (TCM) has played a positive role in tumor treatment. TCM has been confirmed to effectively enhance curative effects and reduce toxic side effects of chemotherapy, palliate clinical syndrome, prevent recurrence and metastasis, improve quality of life and immune function, and prolong survival time [10, 11]. Rutin, a polyphenolic bioflavonoid has shown wide range of pharmacological applications due to its significant antioxidant properties. Conventionally, it is used as antimicrobial, antifungal, and antiallergic agent. However, current research has shown its multispectral pharmacological benefits for the treatment of various chronic diseases such as cancer, diabetes, hypertension, and hypercholesterolemia [12]. Its use is advantageous over other flavonoids as it is a nontoxic and nonoxidizable molecule [13]. In the present study, we demonstrated that rutin suppressed cells proliferation by inducing G2/M cell cycle arrest and promoting apoptosis in LAN-5 cells.

The main biological characteristics of tumor cells are uncontrolled proliferation and higher migration ability. The MTT assay confirmed that rutin significantly suppressed LAN-5 cell proliferation and viability in a concentration-dependent manner. This study show that that rutin-treated LAN-5 cells have a decreased migratory capacity in scratch wound assay and transwell assay. The cell cycle is the series of events that take place in a cell leading to its division and duplication (replication); regulation of the cell cycle involves processes crucial to the survival of a cell. The previous studies reported showed that flavonoids promote cell cycle arrest in distinct phases is main effect on anticancer [14]. Our results indicated that rutin induced G2/M phase arrest in LAN-5 cells. Cell cycle regulation is also important in mediating radiosensitivity. Cells are most sensitive to radiation during the G2/M phase, less sensitive during G1, and least sensitive near the end of the S phase [15]. These results indicate that rutin acts on the G2/M transition checkpoint of the cell cycle. The G2/M phase arrest cells are inclined to radiation-induced apoptosis; therefore, rutin may be a contributing factor in the increased radiosensitivity.

Cancer occurs as the result of a disturbance in the homeostatic balance between cell growth and cell death. Overexpression of antiapoptotic genes and underexpression of proapoptotic genes can result in the lack of cell death that is characteristic of cancer. Apoptosis was the major reason of cell death induced by antitumor drugs and radiosensitization drugs. The B cell lymphoma/leukemia-2 gene (BCL2) and the Bcl2-associated X protein gene (BAX) are an oncogene and a cancer suppressor gene, respectively. In many pathological studies, an unbalanced BCL2/BAX ratio (BCL2/BAX > 1) has been recognized as a signature of the acquisition of apoptosis resistance in cancer cells [16, 17]. Our study show that rutin could induce LAN-5 apoptosis and decrease BCL2 expression and BCL2/BAX ratio. This evidence indicates that the rutin has regulatory role in the BCL2/BAX balance of tumor cells. TNF-*α*, being an endogenous pyrogen, is able to induce fever, apoptotic cell death, cachexia, and inflammation and to inhibit tumorigenesis and viral replication. The present repotted pictogram quantities of TNF-*α* are also produced by the malignant cell of advanced cancer its presence often being associated with poor prognostic factors [18]. In our study, we found that pictogram quantities of TNF-*α* are produced in LAN-5; rutin induced higher TNF-*α* secretion. These results suggest that rutin regulation of *BCL2/BAX* balance and induced high levels of TNF-*α* secretion is a key role in inducing the apoptosis of tumor cell.

The MYCN gene is a member of the MYC family of transcription factors and encodes a protein with a basic helix-loop-helix (bHLH) domain. Deregulated MYC expression is a key event for malignant transformation by inducing multiple tumor-promoting events, including uncontrolled cell proliferation, cell mass expansion, and genomic instability, and contributes to the genesis of a large number of human cancers [19–21]. In neuroblastoma, MYCN amplification occurs in approximately 25% of neuroblastoma cases; MYCN oncogene activation through amplification is an important hallmark of advanced tumor stage and poor prognosis [22]. Importantly, downregulation of MYCN expression results in apoptosis, decreased proliferation, and/or neuronal differentiation in NB cells in vitro [23, 24]. Consequently, MYCN is an attractive target for therapy in high-risk NB. In our study, we found that rutin could down regulate the expression of MYCN in a dose-dependent manner.

In conclusion, our study demonstrated that rutin plays the antiproliferation of human neuroblastoma cells role through inducing G2/M phase cell cycle arrest and triggering apoptosis; this event is a BCL2-independent process. Moreover, rutin could inhibit MYCN expression. Rutin is a potential therapeutic drug for the treatment of neuroblastoma, particularly in MYCN expression tumor.

## Figures and Tables

**Table 1:** The effect of rutin on cell cycle.

Rutin (*μ*M)	G0/G1 (%)	*S* (%)	G2/M (%)
0	48.57 ± 2.24	42.63 ± 2.35	8.8 ± 1.32
25	46.73 ± 1.55	43.22 ± 1.59	10.05 ± 1.48
50	48.71 ± 1.69	36.83 ± 1.21	14.46 ± 1.43^∗^
100	46.87 ± 2.11	33.08 ± 1.04	20.05 ± 2.18**
